# Correlation analysis of surgical outcomes and spino-pelvic parameters in patients with degenerative lumbar scoliosis

**DOI:** 10.3389/fsurg.2023.1319884

**Published:** 2024-01-04

**Authors:** Hang Zhou, Zhancheng Liang, Pengfei Li, Huihong Shi, Anjing Liang, Wenjie Gao, Dongsheng Huang, Yan Peng

**Affiliations:** ^1^ Department of Orthopedics, Sun Yat-sen Memorial Hospital of Sun Yat-sen University, Guangzhou, Guangdong, China; ^2^ Department of Spinal Surgery, Fosun Group, Foshan Fosun Chancheng Hospital, Foshan, Guangdong, China

**Keywords:** degenerative lumbar scoliosis, spino-pelvic parameters, pelvic incidence, lumber lordosis, postoperative quality of life, age

## Abstract

**Objectives:**

The study aims to analyze factors that affect the postoperative health-related quality of life (HRQOL) of degenerative lumbar scoliosis (DLS) patients and explore the appropriate pelvic incidence minus lumbar lordosis (PI-LL) value for Chinese DLS patients.

**Methods:**

DLS patients who met the inclusion and exclusion criteria were included in this study. General information, spino-pelvic parameters, and HRQOL were collected. Correlation analysis was used to explore the spino-pelvic parameters that affect the postoperative HRQOL. Thresholds of each parameter were obtained using the receiver operating characteristic (ROC) curve. Regardless of the effect of age, DLS patients were classified into three groups according to the SRS-Schwab classification: group 0 means PI-LL < 10°, group+means PI-LL = 10–20°, and group ++ means PI-LL > 20°. Postoperative HRQOL was analyzed using variance methods. The ROC curve was used to measure the appropriate PI-LL threshold. When considering the effect of age, the patients with Oswestry Disability Index (ODI) < 75% percentile were considered to have a satisfactory clinical outcome, which was drawn to an equation between PI-LL, age, and PI by multiple linear regression equation.

**Results:**

A total of 71 patients were included. Compared with the control group, there were significant differences in both postoperative ODI and Scoliosis Research Society 22 (SRS-22) scores when the postoperative Cobb angle ≤11°, postoperative lumbar lordosis index (LLI) > 0.8, postoperative sagittal vertical axis (SVA) ≤ 5 cm, postoperative T1 pelvic angle (TPA) ≤ 16° and postoperative global tilt (GT) ≤ 22°, respectively. Regardless of the effect of age, there was a statistical difference in postoperative HRQOL between group 0 and group ++. The PI-LL threshold derived from the ROC curve was 14.4°. Compared with the PI-LL > 14° group, the PI-LL ≤ 14° group achieved a lower postoperative ODI score and a higher postoperative SRS-22 score. Considering the influence of age, the equation for ideal PI-LL was PI-LL = 0.52age + 0.38PI-39.4 (*R* = 0.509, *p* = 0.001).

**Conclusions:**

PI-LL was an important parameter that affects the postoperative HRQOL of DLS patients. Sufficient LL should be restored during the operation (LL ≥ PI-14°). The appropriate PI-LL value was affected by age. Smaller LL needed to be restored as the age increased.

## 1 Introduction

Degenerative lumbar scoliosis (DLS) refers to the asymmetric degeneration of the lumbar discs and facet joints after bone maturation, resulting in coronal scoliosis with a Cobb angle greater than 10° (1). The incidence of DLS was ∼13.3% in the Han population for people over the age of 40 (2). DLS patients often suffer from low back pain and functional limitations. Surgery remains an inevitable choice for DLS patients when conservative treatment fails (3, 4).

In 2012, the effects of spino-pelvic parameters on HRQOL were first reviewed in the SRS-Schwab study (5). Since then, more and more research has shown that there was a correlation between spino-pelvic parameters and surgical outcomes of DLS patients (6–9). Among the spino-pelvic parameters, the study of the ideal PI-LL value was particularly important, because it can provide guidance on how much lordosis should be restored during surgery (10–12).

However, the ideal PI-LL value considerably varies in different populations. According to the SRS-Schwab classification, PI-LL between ±10° is considered to be a suitable range (5). Hai et al. (13) found that the appropriate range of PI-LL for Chinese adult degenerative scoliosis is between 10° and 20°. Qingwei et al. found that the ideal PI-LL range for Chinese DLS patients is between 15° and 28° (14). In addition, age appears to be an independent factor that affects the ideal PI-LL value. Lafage et al. found that the ideal PI-LL threshold is age-related (15, 16). The relationship between age and ideal PI-LL value in Chinese DLS remains unknown.

To this end, this study sought to evaluate the parameters that affect the postoperative HRQOL of DLS patients, explore the appropriate PI-LL range, and clarify the relationship between the PI-LL value and age of Chinese DLS patients.

## 2 Materials and methods

### 2.1 Patient population

This is a single-center, retrospective study. All experiments were carried out in accordance with the ethical standards of the national committees on human experimentation and the Helsinki Declaration of 1964 and later versions. The current research was approved by the Institutional Research Ethical Committee of Sun Yat-sen University (approval number: SYSEC-KY-KS-2020-202). Informed consent was obtained from all subjects and/or their legal guardians. A total of 71 patients (26 males, 45 females, 62.7 ± 7.7 y) with DLS, who were admitted to our hospital from January 2011 to January 2019 and met the inclusion criteria, were included. The inclusion criteria for enrollment were patients aged not less than 45 years at the time of surgery, who had Cobb angle of lumbar curves ≥ 10°, who received lumbar or thoracolumbar fixation surgery in our hospital, who completed preoperative and postoperative radiographic data and functional evaluation results, and who was followed up for ≥ 2 years. The exclusion criteria for the database included a previous history of lumbar fixation surgery, history of scoliosis before adulthood, spinal deformity secondary to ankylosing spondylitis, neuromuscular, vertebral dysplasia syndrome, infections, tumor, or posttraumatic conditions.

## 3 Data collection

**Figure 1:**
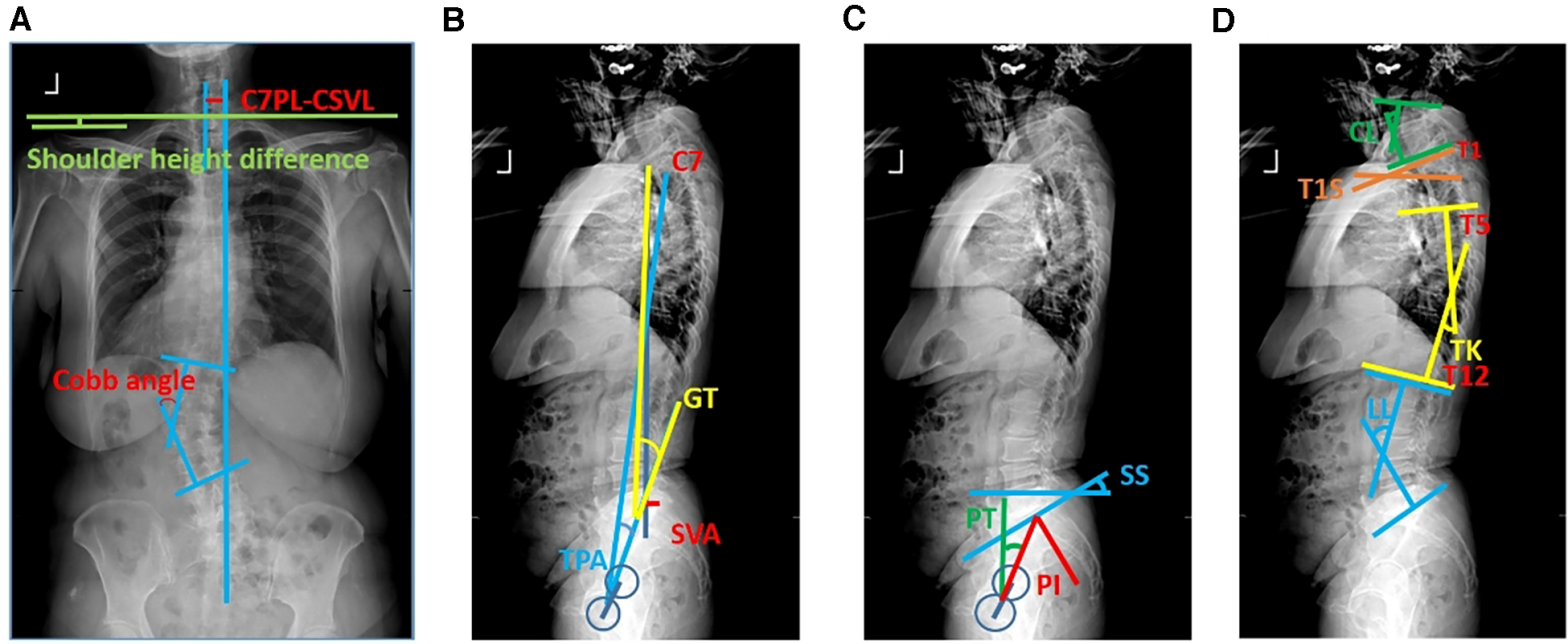
Schematic diagram of spino-pelvic parameters. Schematic diagram of (**A**) coronal plane parameters, (**B**) sagittal plane parameters, (**C**) pelvic parameters, and (**D**) sagittal balance parameters.

The general and surgical information of patients was recorded. General information included age, gender, body mass index (BMI), operation time, intraoperative blood loss, postoperative drainage, and postoperative hospital stay. The spino-pelvic parameters, including Cobb angle, C7PL-central sacral vertical line (CSVL), pelvic incidence (PI), pelvic tilt (PT), sacral slope (SS), lumbar lordosis (LL), thoracic kyphosis (TK), cervical lordosis (CL), T1 slope (T1S), C2-7 sagittal vertical axis (C2-7 SVA), sagittal vertical axis (SVA), T1 pelvic angle (TPA), and global tilt (GT), before surgery and at the last follow-up were measured (Figure 1). Lumbar lordosis distribution index (LDI: L4-S1 angle/L1-S1 angleS1) and lumbar lordosis index (LLI: LL/PI) were calculated. Oswestry Disability Index (ODI), Visual Analogue Scale (VAS), and Scoliosis Research Society 22 (SRS-22) scores were used to evaluate the HRQOL. ODI, VAS, and SRS-22 were collected when patients were first admitted to the hospital (preoperative) and last maximum follow-up (postoperative), respectively.

## 4 Statistical analysis

Statistical analyses were performed using the SPSS 25.0 software. All spino-pelvic parameters and other data were correlated with ODI, VAS, and SRS-22 obtained at the last follow-up. Pearson’s correlation test was used for continuous normally distributed variables, and Spearman’s correlation test was used for continuous non-normal variables. The comparison between the two groups of continuous variables was analyzed by independent sample *t*-test, and the comparison of measurement data between the three groups was analyzed by analysis of variance. The receiver operating characteristic (ROC) curve was used to calculate the maximum Youden index (sensitivity + specificity-1) to find the best threshold. The trend graph between PI-LL and ODI was analyzed using a Loess fitting curve, and the linear equation between PI-LL, PI, and age was obtained by multiple linear regression analysis. A *p* value of <0.05 indicated a statistical difference, and a *p* value of <0.01 indicated a significant statistical difference.

## 5 Results

### 5.1 Characterization of demographic and radiological parameters in DLS patients

A total of 71 DLS cases were included in this study, including 26 males and 45 females, with an average age of 62.7 ± 7.7 years, the youngest being 45 years old, and the oldest being 84 years old. To evaluate the long-term effect of spino-pelvic parameters on the surgical outcomes, postoperative ODI, VAS, and SRS were collected at the last maximum follow-up time. The average follow-up time was 49.2 ± 21.6 months, the average operation time was about 249.0 ± 69.3 min, the average intraoperative blood loss was 639.3 ± 648.9 ml, the average preoperative ODI was 46.5 ± 15.5%, and the average ODI at the last follow-up was 17.1 ± 18.6% (Table 1). The average SRS-22 total score was 91.4 ± 15.2 points at the last follow-up. The spino-pelvic parameters before and after surgery were shown (Table 2).

**Table 1:** Summary of clinical data in DLS patients.

Variables	Mean ± SD	Minimum	Maximum
Age (year)	62.7 ± 7.7	45	84
Follow-up time (month)	49.2 ± 21.6	24	114
Operation time (minute)	249.0 ± 69.3	135	510
Number of fusion segments	2.9 ± 1.8	1	8
Intraoperative blood loss (ml)	639.3 ± 648.9	20	3,400
Postoperative drainage (mL)	814.0 ± 749.1	15	3,400
Postoperative hospital stay (day)	13.6 ± 4.5	5	35
Preoperative ODI (%)	46.5 ± 15.5	18.0	77.8
Last follow-up ODI (%)	17.1 ± 18.6	0	73.3
Last follow-up VAS	2.6 ± 2.7	0	11
Last follow-up SRS-22	91.4 ± 15.2	43	110

**Table 2:** Characterization of spino-pelvic parameters in DLS patients.

Variables	Mean ± SD	Minimum	Maximum
Preoperative Cobb angle (°)	15.6 ± 6.6	10.1	46.9
Preoperative PI (°)	49.5 ± 10.8	25.7	82.8
Preoperative PI-LL (°)	14.5 ± 14.2	−20.3	42.8
Preoperative PT (°)	21.7 ± 10.1	3.7	53.7
Preoperative SS (°)	27.8 ± 10.9	0	50.3
Preoperative LL (°)	34.9 ± 16.2	−4.4	63.4
Postoperative Cobb angle (°)	11.5 ± 7.7	0	43.6
Postoperative PI (°)	48.2 ± 10.9	29.0	84.8
Postoperative PI-LL (°)	13.3 ± 13.8	−19.8	38.3
Postoperative PT (°)	20.0 ± 9.2	0.6	46.3
Postoperative SS (°)	28.4 ± 9.4	0	48.8
Postoperative LL (°)	36.2 ± 14.0	4.5	61.6
Postoperative LDI	0.9 ± 0.6	0.2	4.1
Postoperative LLI	0.7 ± 0.3	0.1	1.6
Postoperative TK (°)	24.6 ± 13.4	−2.2	58.4
Postoperative T1S (°)	21.4 ± 9.4	6.7	50.9
Postoperative CL (°)	18.2 ± 14.4	−13.4	50.1
Postoperative C2-7SVA (cm)	1.6 ± 1.4	−1.6	5.5
Postoperative SVA (cm)	3.6 ± 4.8	−5.8	17.7
Postoperative TPA (°)	17.6 ± 10.7	−7.1	48.5
Postoperative GT (°)	22.5 ± 12.9	−4.1	58.7
Postoperative coronal offset (cm)	1.2 ± 1.3	0	5.5

### 5.2 Correlation between spino-pelvic parameters and surgical outcomes

Spino-pelvic parameters related to the postoperative quality of life included postoperative Cobb angle, postoperative LL, postoperative PI-LL, postoperative LLI, and postoperative sagittal plane balance parameters (SVA, TPA, GT). The parameter with the largest correlation coefficient was postoperative PI-LL. Among them, postoperative Cobb angle, postoperative PI-LL, postoperative PT, postoperative SVA, postoperative TPA, and postoperative GT were positively correlated with postoperative ODI, and the improvement rate of Cobb angle, postoperative LL, and postoperative LLI were negatively correlated with postoperative ODI. There was a positive correlation between VAS and postoperative Cobb angle, postoperative PI-LL, postoperative LLI, and postoperative SVA. The postoperative SRS-22 score showed the same trends as ODI (Table 3). The parameters that showed no correlation with postoperative HRQOL included preoperative Cobb angle, preoperative PI, preoperative PI-LL, preoperative PT, preoperative SS, postoperative PI, postoperative SS, postoperative LDI, postoperative TK, postoperative LL-TK, postoperative T1S, postoperative CL, postoperative C2-7SVA, and postoperative C7PL-CSVL (Table 3).

**Table 3:** Correlation analysis of spino-pelvic parameters and postoperative HRQOL.

Variables	*r* value		
	ODI	VAS	SRS-22
Postoperative Cobb angle	0.309**	0.356**	−0.320**
Postoperative PI-LL	0.398**	0.307**	−0.341**
Postoperative PT	0.294*	0.094	−0.218
Postoperative LL	−0.314**	−0.208	0.272*
Postoperative LLI	−0.389**	0.307**	0.327*
Postoperative SVA	0.279*	0.288*	−0.422**
Postoperative TPA	0.283*	0.167	−0.338*
Postoperative GT	0.303*	0.176	−0.332*

* *p* < 0.05.

** *p* < 0.01.

### 5.3 Threshold of spino-pelvic parameter

**Figure 2:**
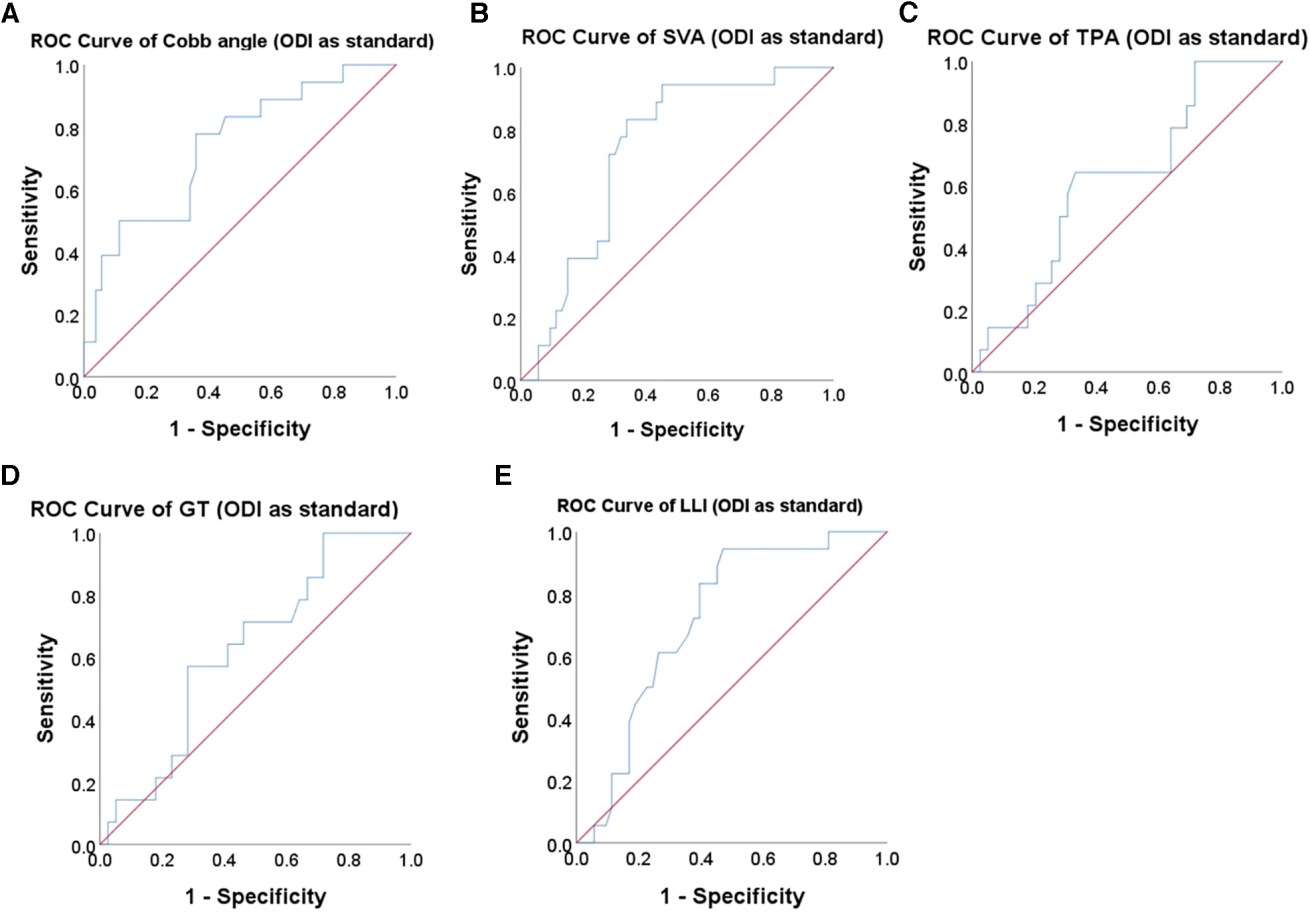
ROC curve to find the optimum cutoff point for spino-pelvic parameters. The ROC curve of (**A**) Cobb angle, (**B**) SVA, (**C**) TPA, (**D**) GT, and (**E**) LLI in predicting the postoperative ODI of DLS patient.

**Figure 3:**
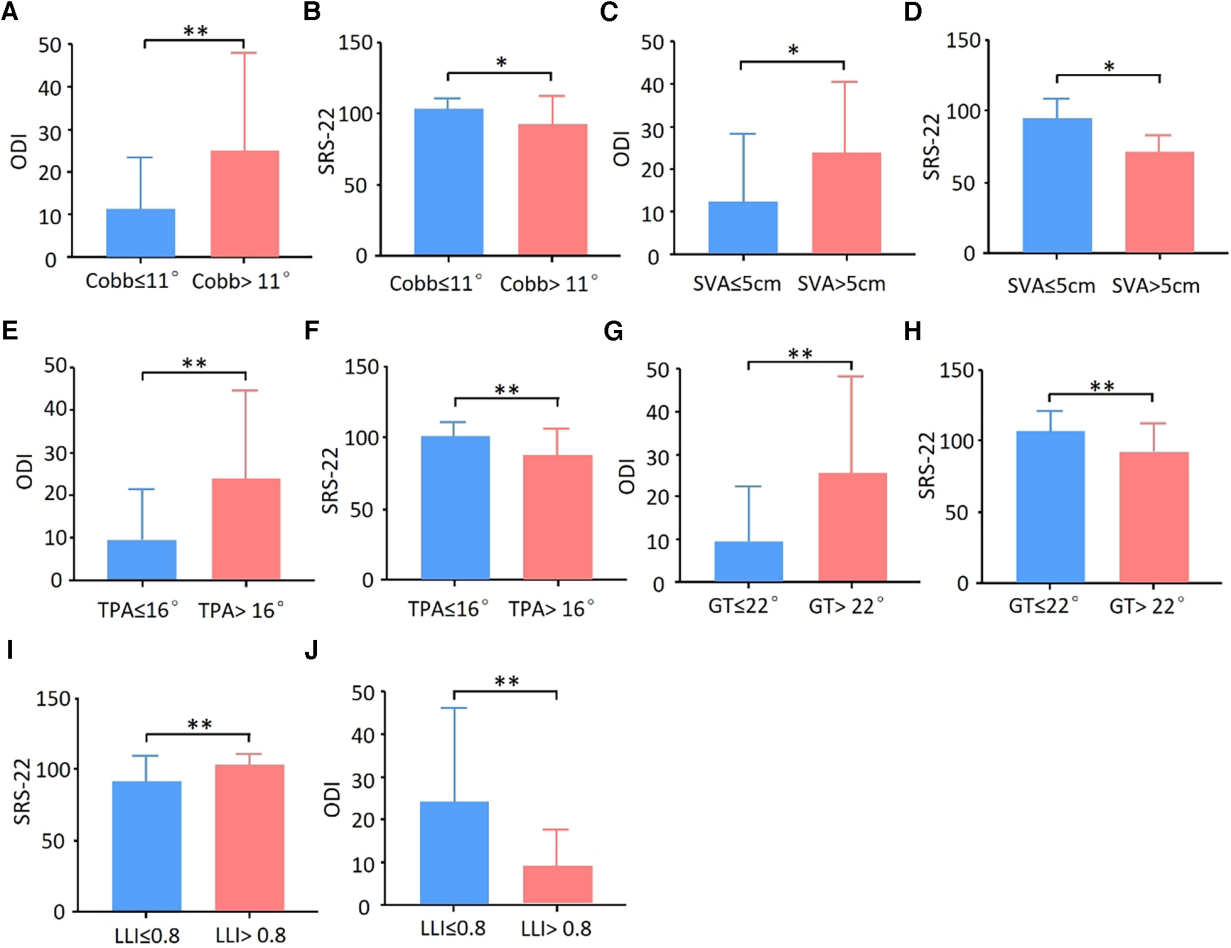
Threshold of spino-pelvic parameters in DLS patients. Using the results of the ROC curve as the thresholds, the postoperative ODI score and SRS-22 score were compared. Better HRQOL could be obtained when (**A,B**) Cobb angle ≤ 11°, (**C,D**) SVA ≤ 5 cm, (**E,F**) TPA ≤ 16°, (**G,H**) GT ≤ 22°, and (**I,J**) LLI >0.8. *p < 0.05, **p < 0.01.

To further analyze how spino-pelvic parameters affect the HRQOL, the thresholds of each parameter were calculated. The postoperative ODI < 75% percentile cutoff value was set as the satisfied clinical effect, and the postoperative ODI was used as the outcome index to establish the ROC curve with the postoperative Cobb angle (Figure 2A). By calculating the maximum value of the Youden index, the best cutoff value of the postoperative Cobb angle was 10.8°. At this time, area under the curve (AUC) = 0.737, sensitivity = 0.778, specificity = 0.642. Taking Cobb angle = 11° as the threshold, DLS patients were divided into two groups: group A, Cobb angle ≤ 11°, group B, Cobb angle > 11°. The postoperative HRQOL was compared between the two groups. The results showed that compared with group B, group A had a significantly lower postoperative ODI score and a higher SRS-22 score. Better postoperative HRQOL could be obtained when the postoperative Cobb angle was ≤11° (Figure 3A, B). Similarly, the thresholds of each parameter were as follows: postoperative SVA ≤ 5 cm (AUC = 0.663, sensitivity = 0.500, specificity = 0.795), postoperative TPA ≤ 16° (AUC = 0.620, sensitivity = 0.643, Specificity = 0.667), postoperative GT ≤ 22° (AUC = 0.627, sensitivity = 0.571, specificity = 0.718), postoperative LLI (LL/PI) > 0.8 (AUC = 0.721, sensitivity = 0.944, specificity = 0.500) (Figures 2B–E, 3C–J).

### 5.4 Explore the optimum PI-LL value in Han DLS

**Figure 4:**
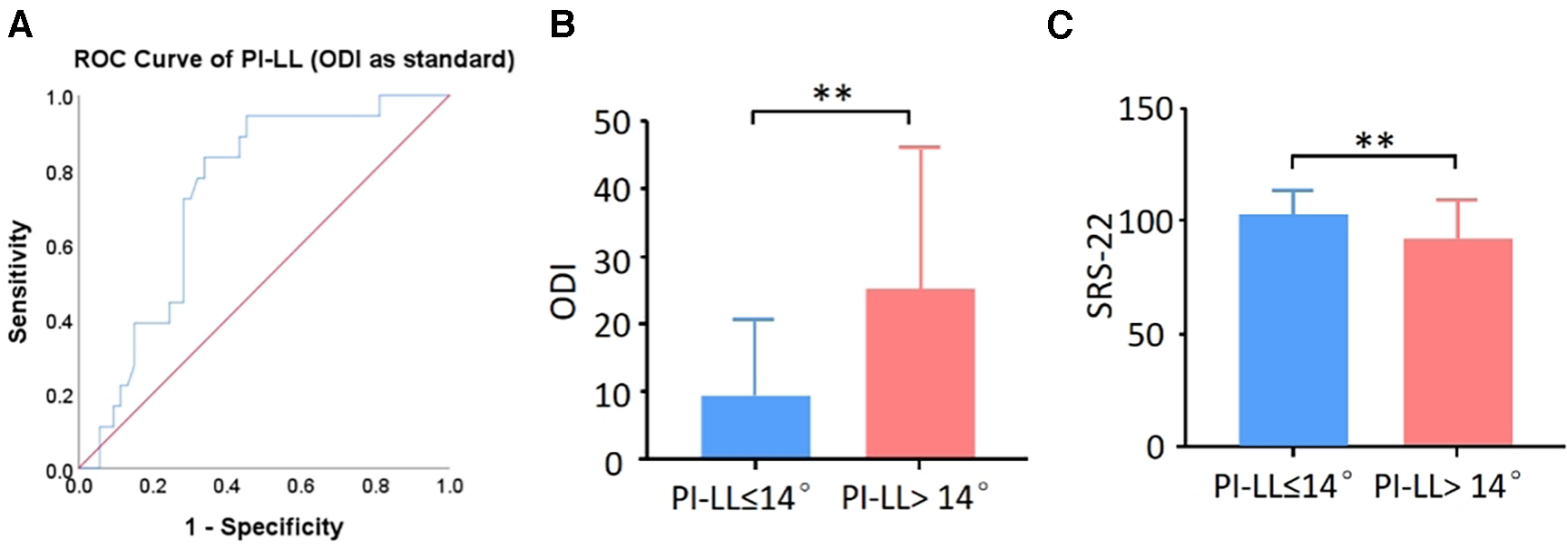
Explore the optimum PI-LL in DLS patients. (**A**) ROC curve of PI-LL in predicting postoperative ODI of DLS patients. (**B**) ODI score and (**C**) SRS-22 score were compared between the PI-LL ≤ 14° group and PI-LL > 14° group. **p < 0.01.

To explore the ideal PI-LL value in our DLS patients, patients were classified into three groups according to the SRS-Schwab classification: group 0, PI-LL < 10°; group +, PI-LL = 10–20°; and group ++, PI-LL > 20°. According to the results, there was a statistical difference in HRQOL score between group 0 and group ++. The postoperative ODI and SRS-22 of group + were better than those of group ++, but there was no statistical difference. The Loess fitting curve showed a significant increasing trend of ODI when the postoperative PI-LL was between 10° and 20°, suggesting that the appropriate threshold for postoperative PI-LL may be between 10° and 20°. The postoperative ODI <75% percentile cutoff value was set as a satisfying clinical effect, and the postoperative ODI was used as the outcome index to establish an ROC curve with postoperative PI-LL. By calculating the maximum value of the Youden index, the best cutoff value of the postoperative PI-LL was 14.4°. At this time, AUC = 0.735, sensitivity = 0.833, and specificity = 0.660 (Figure 4A). DLS patients were divided into group C (PI-LL ≤ 14°) and group D (PI-LL > 14°), and postoperative HRQOL was compared. The results showed that compared with group D, group C showed a significantly lower postoperative ODI score and a higher SRS-22 score (Figure 4B, C). Taken together, these results showed that better postoperative HRQOL could be obtained when PI-LL ≤ 14°.

### 5.5 Correlation between PI-LL value and age

The DLS patients with ODI < 75% percentile (ie ODI < 24.4%) were classified as the satisfied curative effect group, and 53 cases were included. The equations between postoperative PI-LL and PI and age were established through multiple linear regression analysis by using the same method as Hasegawa et al. (17):

PI-LL=0.52age+0.38PI−39.4(R=0.509,P=0.001)

The *p*-values of age coefficient, PI coefficient, and constant were 0.048, 0.000, and 0.023, respectively. According to the equation, there was a positive correlation between postoperative PI-LL and age and PI. It was suggested that as the age increases, the LL that needs to be restored decreases.

## 6 Discussion

In the current study, to explore the correlation between surgical outcomes and spino-pelvic parameters in Chinese DLS patients, a total of 71 DLS patients were included. We found that postoperative Cobb angle, LLI, SVA, TPA, and GT were closely correctly to ODI and SRS-22 score. In our samples, the ideal PI-LL value was PI-LL ≤ 14° regardless of age and PI-LL = 0.52age + 0.38PI-39.4 (*R* = 0.509, *p* = 0.001) when considering the influence of age.

PI-LL value was reported to be closely related to the postoperative quality of DLS patients. In accordance with previous research, we found that DLS patients with a large PI-LL value obtained poor quality of life. It was reported that a large PI-LL value might give rise to adjacent segment degeneration (ASD) (18). With a 1° increase in PI-LL mismatch (preop and postop), the odds of developing ASD requiring surgery increased by 1.3 and 1.4 fold, respectively (11). Taken together, it was necessary to restore sufficient LL during the surgery for DLS patients.

Although more and more researchers started to realize the importance of LL, it remained a controversial topic: what is the ideal LL for different DLS patients? In previous reports, the ideal PI-LL value varies from −10° to 28° in different research (5, 13, 14). In our study, DLS with PI-LL ≤ 14° showed significantly better postoperative quality of life. Two possible reasons might be accountable for the difference in PI-LL value in different studies. Firstly, different races of patients might have different PI-LL values (19). It had been reported that age-matched subjects from the Chinese population had significantly smaller PI and SS than those from the Caucasian population (20). Secondly, the difference in the age of DLS patients in different studies might be another reason. According to the research by Lafage and Hasegawa et al., age was an independent influence factor of ideal LL for DLS. The LL needed to be restored and became smaller as the age got higher (15, 17). Protopsaltis et al. also found that the threshold of PI-LL was affected by age and PI. The older the age, the greater the threshold of PI-LL (21). In our study, we also found that age was an independent influence factor of LL and became smaller as the DLS got older.

There were certain defects in our study. This was a single-center retrospective study, the level of evidence was relatively low, and it was prone to selectivity bias. The immediate postoperative clinical and radiographic data was not collected during the test, making it difficult to analyze how surgical outcomes change over time. Multicenter and large sample studies are needed to further explore the appropriate ideal PL-LL value.

## 7 Conclusion

The postoperative HRQOL was higher when the postoperative Cobb angle ≤11°, the postoperative LLI > 0.8, the postoperative SVA ≤5 cm, the postoperative TPA ≤ 16°, and the postoperative GT ≤ 22°, respectively. Sufficient LL should be restored during the operation (LL ≥ PI-14°). Moreover, the appropriate PI-LL threshold was affected by PI and age. As the age increased, the LL needed to be restored could be reduced.

## Data Availability

The original contributions presented in the study are included in the article/Supplementary Material; further inquiries can be directed to the corresponding authors.
